# Supplementary material for: Patterns of T and B cell responses to Mycobacterium tuberculosis membrane-associated antigens and their relationship with disease activity in rheumatoid arthritis patients with latent tuberculosis infection
Source: PLoS One. 2021 Aug 2;16(8):e0255639. doi: 10.1371/journal.pone.0255639 (PMC8328311; doi:10.1371/journal.pone.0255639)
Supplement: S1 File — (PDF) [file pone.0255639.s001.pdf]

Supporting Information

Patterns of T and B cell responses to *Mycobacterium tuberculosis* membrane-associated antigens and their relationship with disease activity in rheumatoid arthritis patients with latent tuberculosis infection

Shashi Kant Kumar, Suvrat Arya, Ankita Singh, Ramnath Misra, Amita Aggarwal and Sudhir Sinha

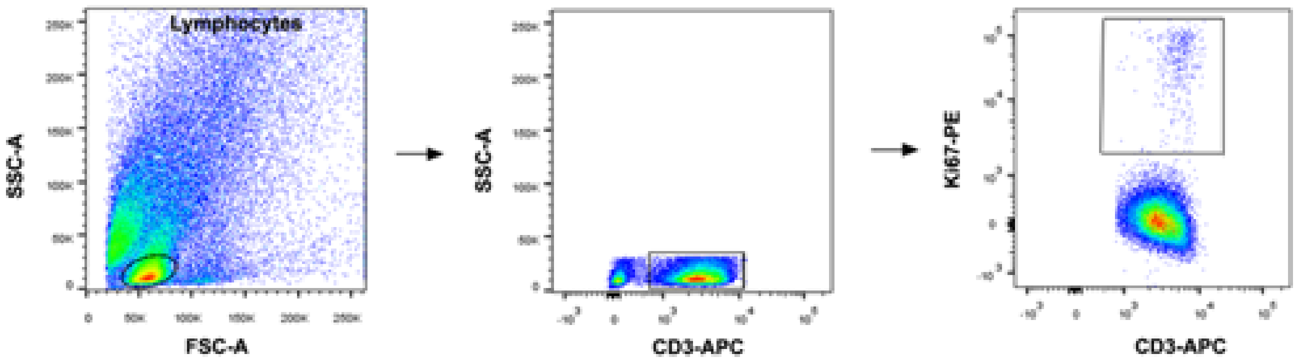

S1 Figure. Gating strategy for T cell proliferation assay

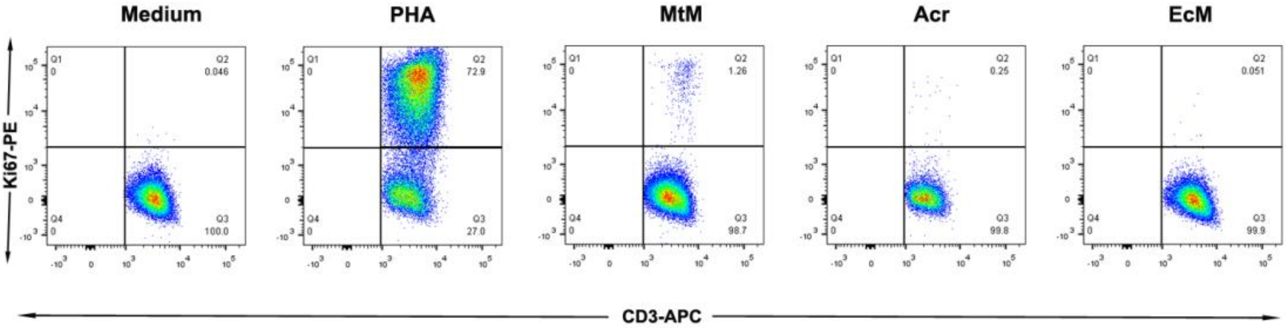

S2 Figure. Representative flow plots for assay controls (medium and PHA) and test antigens (MtM and Acr). In some assays, *Escherichia coli* membrane (EcM) was also used as a 'negative' control.

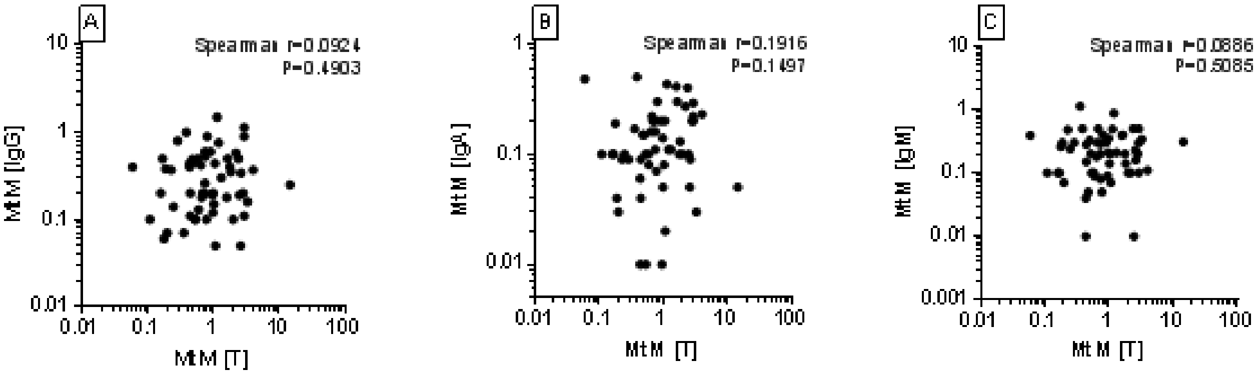

S3 Figure. Correlation between baseline antibody (IgG, IgA or IgM) levels (ΔOD) and T cell proliferative responses (%CD3+Ki67+ cells) against MtM. Corresponding r and P values are shown in the panels (A-C).

S1 Table. Association between high and low responders for T and B cell responses

Data analyzed	HR-B	LR-B	Total
HR-T	9	4	13
LR-T	16	12	28
Total	25	16	41

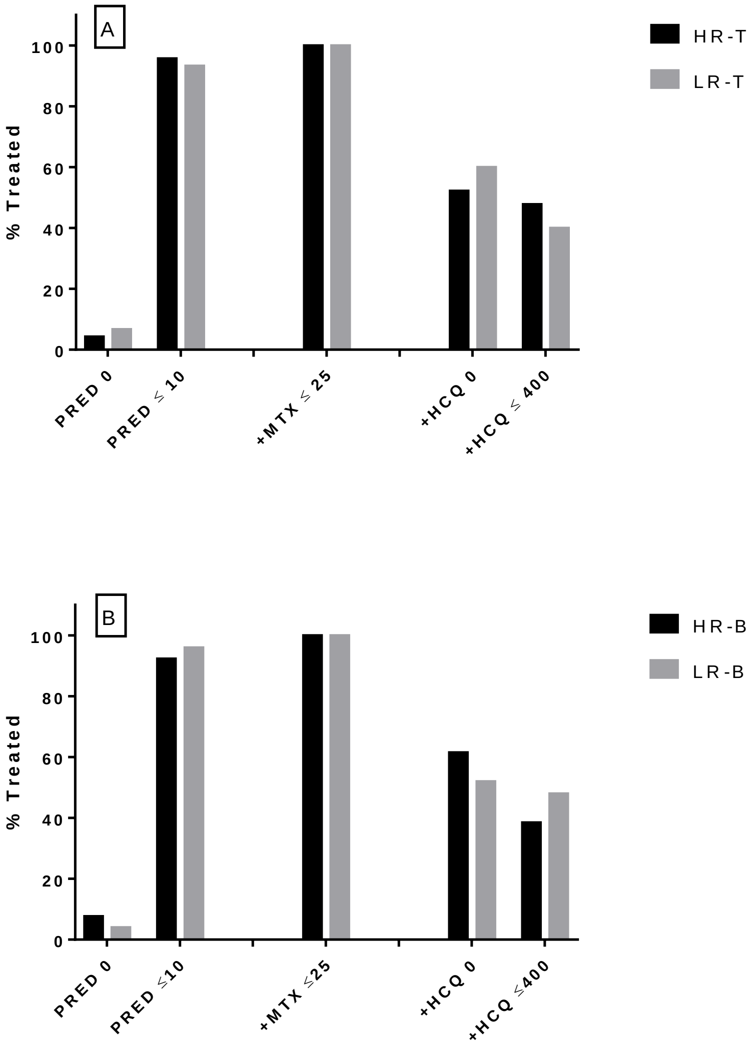

S4 Figure. Effect of treatment on T and B cell responses. Panel A shows proportions of HR-T and LR-T patients treated with following drugs: Prednisolone (0 or up to 10 mg/day, prednisolone + methotrexate (up to 25 mg/week) or prednisolone + methotrexate + hydroxychloroquibne (0 or up to 400 mg/day). Panel B shows proportions of HR-B and LR-B patients treated with the above drug combinations.

S2 Table. Raw data for Fig. 1

Medium	PHA	MtM	Acr
0.15	57.4	3.32	0.23
0.03	92.6	0.44	1.78
0.05	22.2	0.6	0.17
0.32	15.3	1.85	0.27
0.05	20.1	0.51	0.07
0.07	80.7	1.15	0.3
0.25	59	2.96	0.52
0.34	45.7	0.29	0.08
0.02	40.5	0.18	0.02
0.06	39.1	0.36	0.06
0.04	24.1	0.54	0.1
0.01	26.3	0.25	0.28
0.05	81.3	4.05	1.14
0.03	71.1	0.81	0.76
0.04	90.6	1.8	0.71
0.05	85.5	0.65	0.33
0.14	47.5	0.79	0.15
0.18	61.4	0.91	0.26
0.49	81.2	1.03	0.73
0.35	84.8	1.21	0.33
0.25	80.7	0.74	0.42
0.19	91.2	0.45	0.23
0.43	80	0.96	0.58
0.36	87.9	0.69	0.34
0.51	76.1	2.61	0.81
1.05	49.5	1.33	0.99
0.8	81.2	2.23	1.11
0.9	17.7	0.59	1.07
0.09	93.4	2.01	0.7
0.65	78.4	2.84	1.5
0.08	89	0.2	0.08
3.81	89.4	14.6	3.05
0.05	90.5	2.65	1.76
0.28	82.2	0.99	0.11
0.29	63.5	1.08	0.49
0.07	90.1	1.06	1.94
0.03	78.1	2.97	0.56
0.14	96	1.66	1.69
0.44	85.5	0.23	0.44
0.04	20	0.75	0.45
0.28	92.8	0.53	0.12
0.55	40.4	0.19	0.12
0.12	28.2	0.48	0.09
0.15	71.2	0.76	0.56
0.04	41	0.44	0.13
0.11	65	0.06	0.01
0.05	33.6	0.17	0.08
0.06	44.7	0.45	0.17
0.28	80.7	0.11	0.16
0.1	4.73	0.16	0.07
0.14	75.5	1.61	1.11
0.29	99	2.5	0.53
0.12	86.2	0.75	0.32

0.01	71.3	0.39	0.04
0.13	72.8	0.67	0.03
0.18	22.4	2.97	0.3
0.39	23.5	1	0.36
0.06	40.7	2.42	0.65

S3 Table. Raw data for Fig 2

MtM [G]	MtM [A]	MtM [M]	Acr [G]	Acr [A]	Acr [M]
0.16	0.03	0.34	-0.02	-0.03	-0.04
0.44	0.06	0.04	0.37	0	-0.03
0.13	0.08	0.09	-0.01	-0.03	-0.01
0.35	0.13	0.14	0	0.14	0.1
0.45	0.15	0.33	0.16	-0.02	0.2
1.49	0.43	0.49	0.08	0.06	0.11
1.14	0.29	0.3	-0.01	0.11	0.12
0.8	0.09	0.3	0	0.03	-0.01
0.06	0.19	0.26	0	-0.02	-0.01
0.07	0.17	1.12	0	-0.01	-0.02
0.1	0.01	0.1	0.02	0.01	0.07
0.14	0.1	0.24	-0.02	0	-0.04
0.37	0.23	0.11	0.02	0.04	0.05
0.9	0.3	0.4	0.43	-0.02	0
0.4	0.1	0.2	-0.01	0	0
0.42	0.16	0.18	0.09	0.04	0.02
0.1	0.07	0.05	0.01	-0.03	-0.01
0.6	0.2	0.3	0	-0.02	-0.01
0.15	0.08	0.14	-0.01	0	-0.02
0.76	0.11	0.87	-0.04	-0.02	-0.05
0.2	0.2	0.2	0	-0.01	0
0.2	0.09	0.15	0	-0.03	-0.05
0.19	0.01	0.09	0.1	0.06	0.09
0.2	0.2	0.5	-0.02	0.04	-0.09
0.05	0.05	0.23	0.05	0.11	0.04
0.3	0.11	0.21	0.01	-0.01	-0.01
0.58	0.27	0.1	0.1	0.03	0.04
0.5	0.1	0.1	-0.01	-0.02	-0.04
0.1	0.1	0.1	-0.04	0	-0.03
0.2	0.2	0.2	-0.07	0.02	-0.1
0.07	0.03	0.07	-0.01	-0.02	-0.08
0.25	0.05	0.31	-0.01	0	-0.04
0.34	0.09	0.16	0.02	0.06	-0.11
0.12	0.14	0.35	0	0.01	-0.01
0.05	0.02	0.07	-0.05	0.06	-0.1
0.44	0.2	0.2	0.01	0	0.01
0.11	0.22	0.1			
0.5	0.3	0.4	0.02	-0.02	0
0.37	0.09	0.48	0.02	0	-0.03
0.26	0.11	0.08	0.03	-0.03	0
0.1	0.1	0.2	-0.01	-0.02	-0.01
0.38	0.04	0.3	0	-0.01	0.01
0.49	0.15	0.05	0.04	-0.01	0
0.53	0.16	0.08	0.2	0.01	0.03
0.4	0.01	0.01	-0.01	-0.01	-0.01
0.4	0.48	0.39			
0.5	0.1	0.1	0.01	-0.02	-0.06
0.11	0.04	0.28	-0.02	-0.01	-0.01
0.1	0.1	0.1	-0.1	0	-0.11
0.2	0.1	0.1	0	0.04	-0.11
0.18	0.41	0.39	0.01	-0.01	-0.04
0.19	0.1	0.01	0	0.01	-0.06
0.6	0.2	0.29	-0.01	-0.05	-0.06
1	0.5	0.5	0.03	0.01	0

0.18	0.22	0.3	0	-0.01	0.01
0.9	0.2	0.5	0.06	-0.02	-0.04
0.2	0.05	0.21	0	0.01	0
0.5	0.4	0.5	0.14	0	0.01

S4 Table. Raw data for Fig. 3A

MtM-Hi-6	MtM-Hi-12	MtM-Hi-18	MtM-Lo-6	MtM-Lo-12	MtM-Lo-18	Acr-Hi-6	Acr-Hi-12	Acr-Hi-18	Acr-Lo-6	Acr-Lo-12	Acr-Lo-18
16.48	6.11		0.35	0.8		0.34	0.63		0.33	0.17	
6.2			0.01	0.09		7.53			0.38	0.29	0.78
0.23	0.7	1.07	0.27	0.24	1.52	0.41	0.7	5.59	0.31	0.17	0.62
2.39	0.44		0.2	0.17	0.38	12	97		0.36	0.58	1.39
2.33	0.35	0.3	0.04	0.58		3.1	0.3	4.8	0.33	0.4	
0.2	3.3	1.17	0.08	0.85	0.42	0.75	0.68	0.16	0.04	0.46	0.15
2.88		0.47	0.45			0.57		0.09	0.25		
1.9	0.35		0.24	0.18		3.4	18.3		0.31	0.33	
1.52		2.37	0.09			0.41		0.6	0.15		
0.95	5.48		0.13	0.79	0.28	0.8	11.8		0.14	0.32	0.2
0.07	13.61		1.61		0.76	0.08	5.1		0.16		0.93
0.21	20.6	0.99	0.96			0.09	3.49	0.44	0.68		
1.2	4.06	3.25	0.36	0.17		2.2	3	3.7	0.49	0.07	
1.15	7.9		0.3	0.73	0.68	4	32.6		0.23	1.31	1.31
0.67	1.79	11.92	0.78	1		0.7	0.02	1.25	0.95	1.86	
	3.83				1.91		7				0.39
1.18		8.18				0.9		8.3			
6.09	1.55	0.29				0.63	0.25	1.88			
8.19	1.44	50.6				1.1	2.1	17.9			
2.51	4.09					0.45	0.31				
3.62		52.56				1.8		41.5			
0.9	0.74	1.43				5.03	0.73	0.97			
1.91		5.52				7.58		1.28			
13.09		1.22				3.39		1.43			
2.55	1.22	1.15				4.13	0.38	1.75			

S5 Table. Raw data for Fig 3B (IgG)

IgG-Hi-6	IgG-Hi-12	IgG-Hi-18	IgG-Lo-6	IgG-Lo-12	IgG-Lo-18
0.45	0.16		1.08		
0.77	0.89	2.03	0.44	0.07	0.26
1.67	3.83		0.93	0.12	0.84
0.92	0.85	0.85			0.8
0.41	0.35	0.27	0.83	1.17	1.07
0.8			1.3	1.6	
1.05	1.26				0.42
1.43	1.42		1.13		
1	1.4	1.2	1.2	1.8	1
4.77		4.46	0.61		0.43
1.14	1.18				1.5
		0.2			0.85
1.44		2.06	0.92	0.83	
			0.55		
					1.92
					1
			0.25	0.32	0.15
					0.69
				1.4	
					1.28
					0.53
					0.41
					0.47
			0.7		1.45
			0.66	0.66	0.21
			0.69		1.55
					1.89
					0.87

S6 Table. Raw data for Fig. 3B (IgA)

IgA-Hi-6	IgA-Hi-12	IgA-Hi-18	IgA-Lo-6	IgA-Lo-12	IgA-Lo-18
1.5	0.83		1		
0.38	0.92	1.08	0.35	0.07	0.23
0.84	1.47		0.71	0.04	0.61
7	3	2.5			1
3.7	2.26	2.43	0.38	0.25	0.25
2.88			0.85	1.29	
9	7				0.62
1.33	0.67		1.67		
2	3	4.5	1.6	1.8	1.4
2.18		2	1		0.86
0.73	0.53				0.76
		0.75			1.78
0.51		0.61	0.93	0.64	
			0.14		
					0.14
					0.7
			0.13	0.38	0.06
					1.8
				0.73	
					0.7
					0.2
					0.15
					0.71
			1.4		1.8
			0.76	0.32	0.38
			0.45		1.79
					0.08
					0.82

S7 Table. Raw data for Fig. 3B (IgM)

IgM-Hi-6	IgM-Hi-12	IgM-Hi-18	IgM-Lo-6	IgM-Lo-12	IgM-Lo-18
4.75	2		1.44		
0.57	0.64	0.93	0.37	0.1	0.29
0.85	1.23		0.24	0.02	0.17
5	3.17	2.83			1.23
1.09	1	0.55	0.22	0.17	0.17
3.21			1.4	1.8	
1.44	1.33				0.22
2	1.14		0.56		
0.86	1	2.86	0.87	1.26	0.87
1.38		1.63	0.77		1.85
2	1.8				1.41
		2.8			1.38
0.41		1.07	0.91	0.2	
			1.5		
					1.46
					1.19
			0.38	0.5	0.5
					0.25
				1.1	
					1.13
					0.91
					0.27
					0.49
			1.1		1.14
			0.62	0.22	0.11
			1.28		1.2
					0.78
					1.05

S8 Table. Raw data for Fig. 4A

HR-T [BL]	LR-T [BL]	HR-T [F1]	LR-T [F1]	HR-T [F2]	LR-T [F2]
7.24	5.71	4.98	4.81	4.81	3.81
6.26	6.51	4.94	4.91	4.72	4.65
6.24	3.63	5.07	2.21	5.14	2.1
6.92	6.24	5.47	4.98	5.23	4.74
5.59	8.47	4.28	5.01	3.64	5.07
6.87	6.48	5.67	6.48	5.43	5.64
7.9	6.75	4.97	5.04	4.74	4.31
7.23	4.83	6.05	3.23	4.88	2.64
6.98	4.65	4.77	2.95	5.04	2.73
5.99	5.3	5.01	4.28	4.95	3.54
7.63	7.17	5.6	5.35	5.5	4.58
5.07	4.47	4.17	4.09	3.99	3.59
7.36	5.86	4.47	4.76	5.5	4.26
5.81	4.34	4.86	3.99	4.83	3.68
6.39	4.95	5.49	4.82	4.81	4.5
7.7	6.49	4.8	4.89	4.93	5.16
6.71		4.94		4.88	
7.55		6.07		5.34	
7.16		5.41		4.88	
7.31		5.47		4.81	
5.77		5.14		4.72	
5.67		4.99		4.73	
7.16		4.93		4.82	

S9 Table. Raw data for Fig. 4B

HR-T	LR-T
1	6
5	7
5	15
6	15
1.5	6
5	10
0.5	2
2	5
6	4
0.67	2
2.5	10
1.5	0.25
12	4
12	9
0.5	8
1	8
1	
0.08	
6	
2	
6	
12	
5	

S10 Table. Raw data for Fig. 4C

HR-B [BL]	LR-B [BL]	HR-B [F1]	LR-B [F1]	HR-B [F2]	LR-B [F2]
7.24	6.26	4.98	4.94	4.81	4.72
6.24	5.71	5.07	4.81	5.14	3.81
6.92	6.87	5.47	5.67	5.23	5.43
5.59	3.63	4.28	2.21	3.64	2.1
6.51	6.24	4.91	4.98	4.65	4.74
6.75	8.47	5.04	5.01	4.31	5.07
4.83	6.48	3.23	6.48	2.64	5.64
7.23	4.65	6.05	2.95	4.88	2.73
7.63	5.3	5.6	4.28	5.5	3.54
4.34	7.17	3.99	5.35	3.68	4.58
5.81	4.47	4.86	4.09	4.83	3.59
7.55	7.9	6.07	4.97	5.34	4.74
7.31	6.98	5.47	4.77	4.81	5.04
	5.99		5.01		4.95
	5.86		4.76		4.26
	5.07		4.17		3.99
	7.36		4.47		5.5
	6.39		5.49		4.81
	4.95		4.82		4.5
	7.7		4.8		4.93
	6.71		4.94		4.88
	7.16		5.41		4.88
	5.77		5.14		4.72
	5.67		4.99		4.73
	7.16		4.93		4.82
	6.49		4.89		5.16

S11 Table. Raw data for Fig 4D.

HR-B	LR-B
1	5
5	6
6	5
1.5	15
7	15
2	6
5	10
2	4
2.5	2
9	10
12	0.25
0.08	0.5
2	6
	0.67
	4
	1.5
	12
	0.5
	8
	1
	1
	6
	6
	12
	5
	8
