# Supplementary material for: Patterns of T and B cell responses to Mycobacterium tuberculosis membrane-associated antigens and their relationship with disease activity in rheumatoid arthritis patients with latent tuberculosis infection
Source: PLoS One. 2021 Aug 2;16(8):e0255639. doi: 10.1371/journal.pone.0255639 (PMC8328311; doi:10.1371/journal.pone.0255639)
Supplement: Attachment — Submitted filename: Review final. Comments..docx [file pone.0255639.s002.docx]

In this manuscript, Shashi Kant Kumar, et al investigated the immune response to Mtb membrane antigens in RA patients with LTBI. The study is interesting and as well the results also.

However, the following are some concerns:

Different microbial agents are involved as triggers factors in RA, and TLR signalling is extremely important in host defence. In order to complete the immunopathology of RA and to better understand the molecular pathways triggered by Tb infection, the authors are invited to explain in more detail this signalling. In addition, recent in vivo studies identified the importance of Interferon Regulatory Factor 5 (IRF5) in the pathogenesis of RA as a new link between the pathogenic activation of RNA-sensing Toll-like receptors and proinflammatory cytokine production in inflamed joints of arthritic mice. Other than that, in human studies has been found a strong immune response against IRF5 suggesting IRF5 as a target of immune response in RA.

Some articles below are suggested that will certainly give more strength to the work enriching the content.

- Almuttaqi H et al. Advances and challenges in targeting IRF5, a key regulator of inflammation. FEBS J. 2019 May;286(9):1624-1637. doi: 10.1111/febs.14654. Epub 2018 Sep 21.
- Bo M et al. Antibody response to homologous epitopes of Epstein-Barr virus, *Mycobacterium avium subsp. paratuberculosis* (MAP) and IRF5 in patients with different connective tissue diseases and in mouse model of antigen-induced arthritis. J Transl Autoimmun. 2020 Mar 17;3:100048. doi: 10.1016/j.jtauto.2020.100048. eCollection 2020.
- Duffau P et al. Promotion of Inflammatory Arthritis by Interferon Regulatory Factor 5 in a Mouse Model. Arthritis Rheumatol. 2015 Dec;67(12):3146-57. doi: 10.1002/art.39321.
- Bo M et al. Interferon regulatory factor 5 is a potential target of autoimmune response triggered by Epstein-barr virus and *Mycobacterium avium s*ubsp*. paratuberculosis* in rheumatoid arthritis: investigating a mechanism of molecular mimicry. Clin Exp Rheumatol. 2018 May-Jun;36(3):376-381. Epub 2018 Jan 15.

Also, the authors have mentioned the role played by others Mycobacteria in RA, and appears interesting the role played by *Mycobacterium avium* subsp. *paratuberculosis* as a possible trigger factor recently discovered in the aetiology of RA. It would better give more details writing a short and concise paragraph on the role of Mycobacterial infections in RA.

Finally, a recent review on the role of Mycobacterial infections in RA is suggested below in order to complete your manuscript.

-	Bo M et al. Role of Infections in the Pathogenesis of Rheumatoid Arthritis: Focus on Mycobacteria. Microorganisms. 2020 Sep 23;8(10):1459. doi: 10.3390/microorganisms8101459.

Best wishes
